# Persistence of Influenza H5N1 and H1N1 Viruses in Unpasteurized Milk on Milking Unit Surfaces

**DOI:** 10.3201/eid3008.240775

**Published:** 2024-08

**Authors:** Valerie Le Sage, A.J. Campbell, Douglas S. Reed, W. Paul Duprex, Seema S. Lakdawala

**Affiliations:** University of Pittsburgh, Pittsburgh, Pennsylvania, USA (V. Le Sage, D.S. Reed, W.P. Duprex);; Emory University School of Medicine, Atlanta, Georgia, USA (A.J. Campbell, S.S. Lakdawala)

**Keywords:** influenza, H5N1, H1N1, viruses, zoonoses, respiratory infections, cattle, milk, virus stability, United States

## Abstract

Examining the persistence of highly pathogenic avian influenza A(H5N1) from cattle and human influenza A(H1N1)pdm09 pandemic viruses in unpasteurized milk revealed that both remain infectious on milking equipment materials for several hours. Those findings highlight the risk for H5N1 virus transmission to humans from contaminated surfaces during the milking process.

Highly pathogenic avian influenza A(H5N1) virus was detected in US domestic dairy cattle in late March 2024, after which it spread to herds across multiple states and resulted in at least 3 confirmed human infections (*1*). Assessment of milk from infected dairy cows indicated that unpasteurized milk contained high levels of infectious influenza virus (*2*; L.C. Caserta et al., unpub. data, https://doi.org/10.1101/2024.05.22.595317). Exposure of dairy farm workers to contaminated unpasteurized milk during the milking process could lead to increased human H5 virus infections. Such infections could enable H5 viruses to adapt through viral evolution within humans and gain the capability for human-to-human transmission.

The milking process is primarily automated and uses vacuum units, commonly referred to as clusters or claws, which are attached to the dairy cow teats to collect milk (Figure 1) (*3*). However, several steps in the milking process require human input, including forestripping, whereby workers manually express the first 3–5 streams of milk from each teat by hand. Forestripping stimulates the teats for optimal milk letdown, improves milk quality by removing bacteria, and provides an opportunity to check for abnormal milk. The forestripping process can result in milk splatter on the floor of the milking parlor and surrounding equipment and production of milk aerosols. 

**Figure 1:**
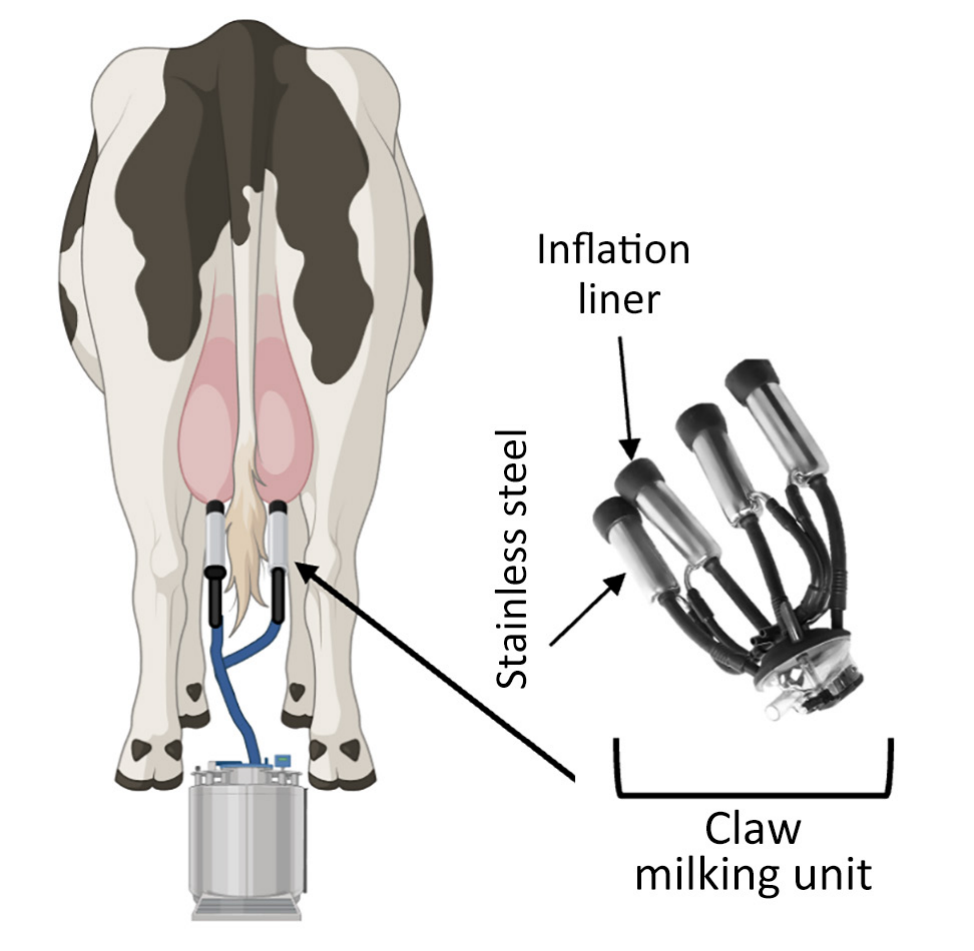
Illustration of milking unit surfaces tested in a study of persistence of influenza H5N1 and H1N1 viruses in unpasteurized milk. Before attaching the milking unit (claw), a dairy worker disinfects the teat ends, performs forestripping of each teat to detect abnormal milk, and then wipes each teat with a clean dry towel. Workers then attach the milking unit to the cow teats. A pulsation system opens and closes the rubber inflation liner (at left) around the teat to massage it, mimicking a human stripping action. A vacuum pump is controlled by a variable speed drive and adjusts the suction to allow milk to flow down a pipeline away from the cow into a bulk tank or directly onto a truck. Additional sources of exposure to humans include handling of raw unpasteurized milk collected separately from sick cows or during the pasteurization process. Schematic created in BioRender (https://www.biorender.com).

After forestripping, each teat is cleaned and dried by hand before the claw is installed. During milking, a flexible rubber inflation liner housed within the stainless-steel shell of the claw opens to enable the flow of milk and closes to exert pressure on the teat to stop the flow of milk (Figure 1). When the flow of milk decreases to a specific level, the claw automatically releases (*3*), at which point residual milk in the inflation liner could spray onto dairy workers, equipment, or the surrounding area. Of note, milking often takes place at human eye level; the human workspace is physically lower than the cows, which increases the potential for infectious milk to contact human workers’ mucus membranes. No eye or respiratory protection is currently required for dairy farm workers, but recommendations have been released (*4*).

 Influenza virus persistence in unpasteurized milk on surfaces is unclear, but information on virus persistence is critical to understanding viral exposure risk to dairy workers during the milking process. Therefore, we analyzed the persistence of infectious influenza viruses in unpasteurized milk on surfaces commonly found in milking units, such as rubber inflation liners and stainless steel (Figure 1). 

For infectious strains, we used influenza A(H5N1) strain A/dairy cattle/TX/8749001/2024 or a surrogate influenza A(H1N1)pdm09 pandemic influenza virus strain, A/California/07/2009. We diluted virus 1:10 in raw unpasteurized milk and in phosphate-buffered saline (PBS) as a control. As described in prior studies (*5*–*7*), we pipetted small droplets of diluted virus in milk or PBS onto either stainless steel or rubber inflation liner coupons inside an environmental chamber. We then collected virus samples immediately (time 0) or after 1, 3, or 5 hours to detect infectious virus by endpoint titration using a 50% tissue culture infectious dose assay (*7*). To mimic environmental conditions within open-air milking parlors in the Texas panhandle during March–April 2024, when the virus was detected in dairy herds, we conducted persistence studies using 70% relative humidity. 

We observed that the H5N1 cattle virus remained infectious in unpasteurized milk on stainless steel and rubber inflation lining after 1 hour, whereas infectious virus in PBS fell to below the limit of detection after 1 hour (Figure 2, panel A). That finding indicates that unpasteurized milk containing H5N1 virus remains infectious on materials within the milking unit. To assess whether a less pathogenic influenza virus could be used as a surrogate to study viral persistence on milking unit materials, we compared viral decay between H5N1 and H1N1 in raw milk over 1 hour on rubber inflation liner and stainless-steel surfaces (Figure 2, panel B). The 2 viruses had similar decay rates on both surfaces, suggesting that H1N1 can be used as a surrogate for H5N1 cattle virus in studies of viral persistence in raw milk. Further experiments examining H1N1 infectiousness over longer periods revealed viral persistence in unpasteurized milk on rubber inflation liner for at least 3 hours and on stainless steel for at least 1 hour (Figure 2, panel C). Those results indicate that influenza virus is stable in unpasteurized milk and that influenza A virus deposited on milking equipment could remain infectious for >3 hours.

**Figure 2:**
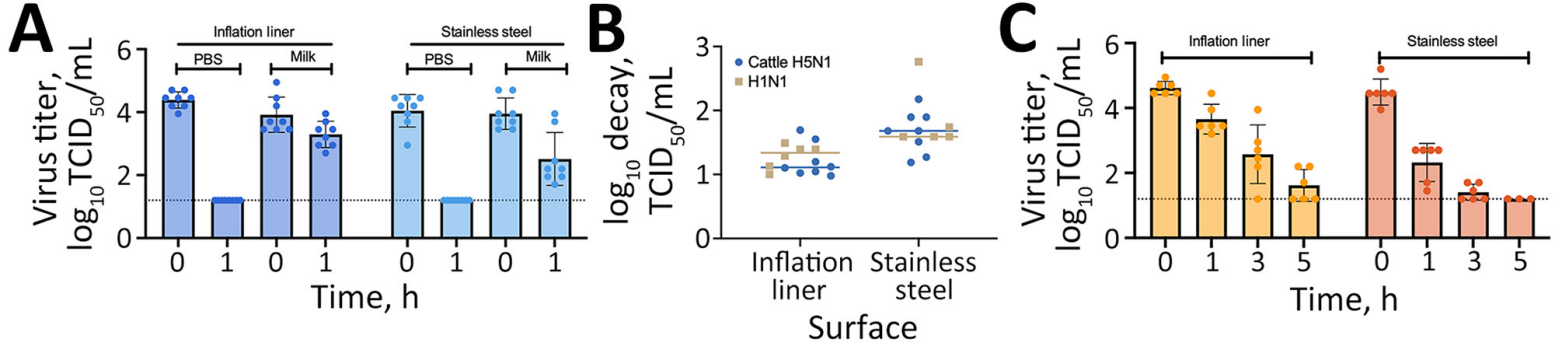
Viral titers in a study of persistence of influenza H5N1 and H1N1 viruses in unpasteurized milk on milking unit surfaces. A) Viral titers of bovine A(H5N1) virus diluted 1:10 in unpasteurized milk or PBS and deposited as ten 1-μL droplets onto the indicated surfaces. Droplets were recovered immediately after deposition (time 0) or after 1 hour of aging at 70% relative humidity (RH) at 21°C. Colored dots indicate measurements for each droplet; error bars indicate SD. Horizontal dotted lines indicate the theoretical limits of detection. B) Comparison of log decay values of H5N1 and H1N1 viruses in unpasteurized milk at 70% RH for 1 hour on rubber inflation liners and stainless steel. Decay was calculated as a ratio of the viral titer at time 0 divided by the titer after 1 hour. Colored symbols indicate measurements for each droplet. Horizonal lines indicate median values. C) Viral titers of the H1N1 virus diluted 1:10 in unpasteurized milk on the 2 surfaces at 70% RH for 0, 1, 3, or 5 hours at 23.6°C–25°C. Each symbol is a replicate of >2 biologic replicates using 2 distinct lots of unpasteurized milk performed in triplicate. Virus titer was calculated using the traditional TCID_50_ assay on MDCK cells. Colored dots indicate measurements for each droplet; error bars indicate SD. Horizontal dotted lines indicate the theoretical limits of detection. All raw data are available at https://doi.org/10.6084/m9.figshare.c.7242034.v1. PBS, phosphate buffered saline; TCID_50_, 50% tissue culture infectious dose.

Taken together, our data provide compelling evidence that dairy farm workers are at risk for infection with H5N1 virus from surfaces contaminated during the milking process. To reduce H5N1 virus spillover from dairy cows to humans, farms should implement use of personal protective equipment, such as face shields, masks, and eye protection, for workers during milking. In addition, contaminated rubber inflation liners could be responsible for the cattle-to-cattle spread observed on dairy farms. Sanitizing the liners after milking each cow could reduce influenza virus spread between animals on farms and help curb the current outbreak.
